# Supplementary figures and images for: Citation proximus: The role of social and semantic ties on citations
Source: PLoS One. 2025 Oct 27;20(10):e0335366. doi: 10.1371/journal.pone.0335366 (PMC12558470; doi:10.1371/journal.pone.0335366)

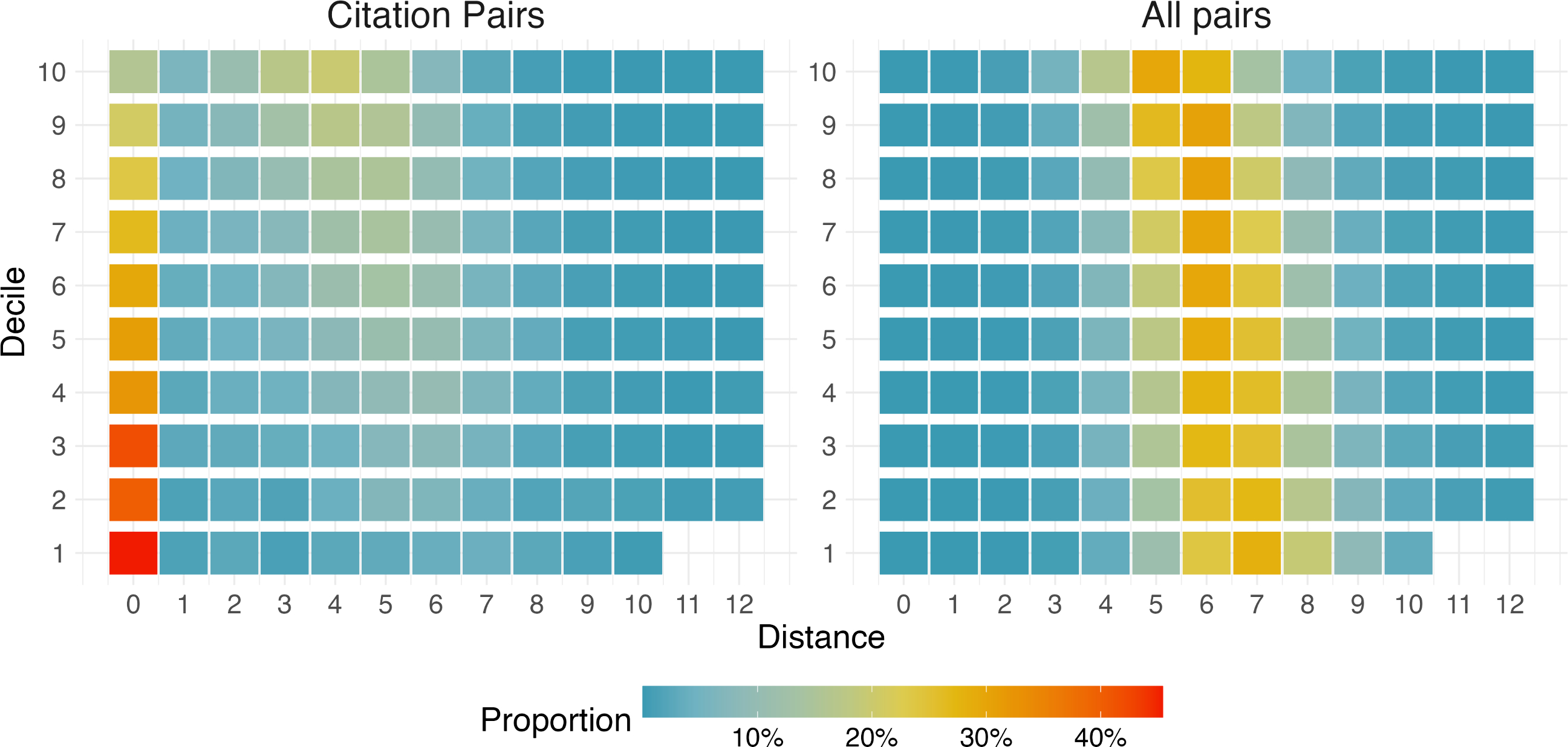

Supplement: S1 Fig — (TIF) [file pone.0335366.s001.tif]

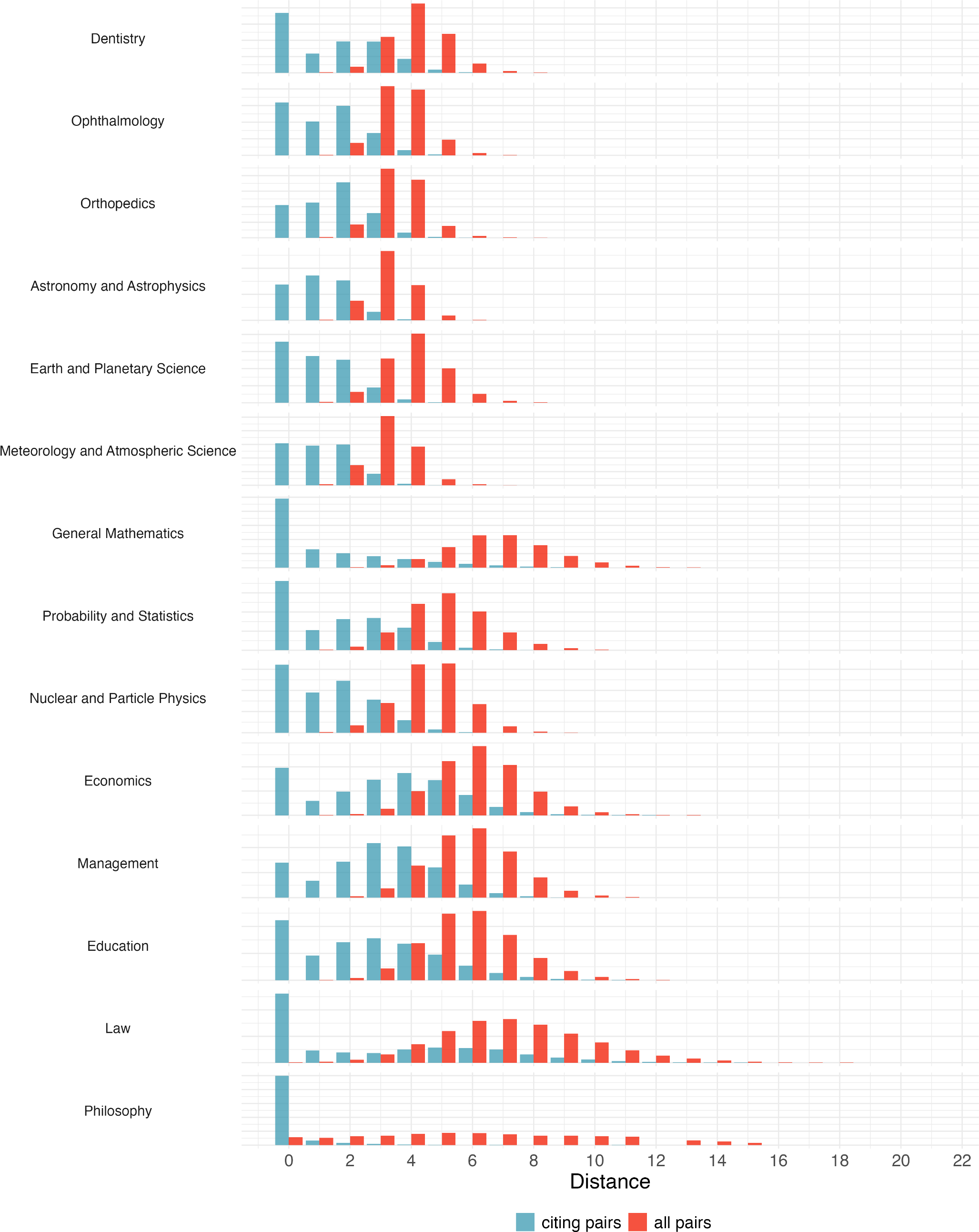

Supplement: S2 Fig — (TIF) [file pone.0335366.s002.tif]

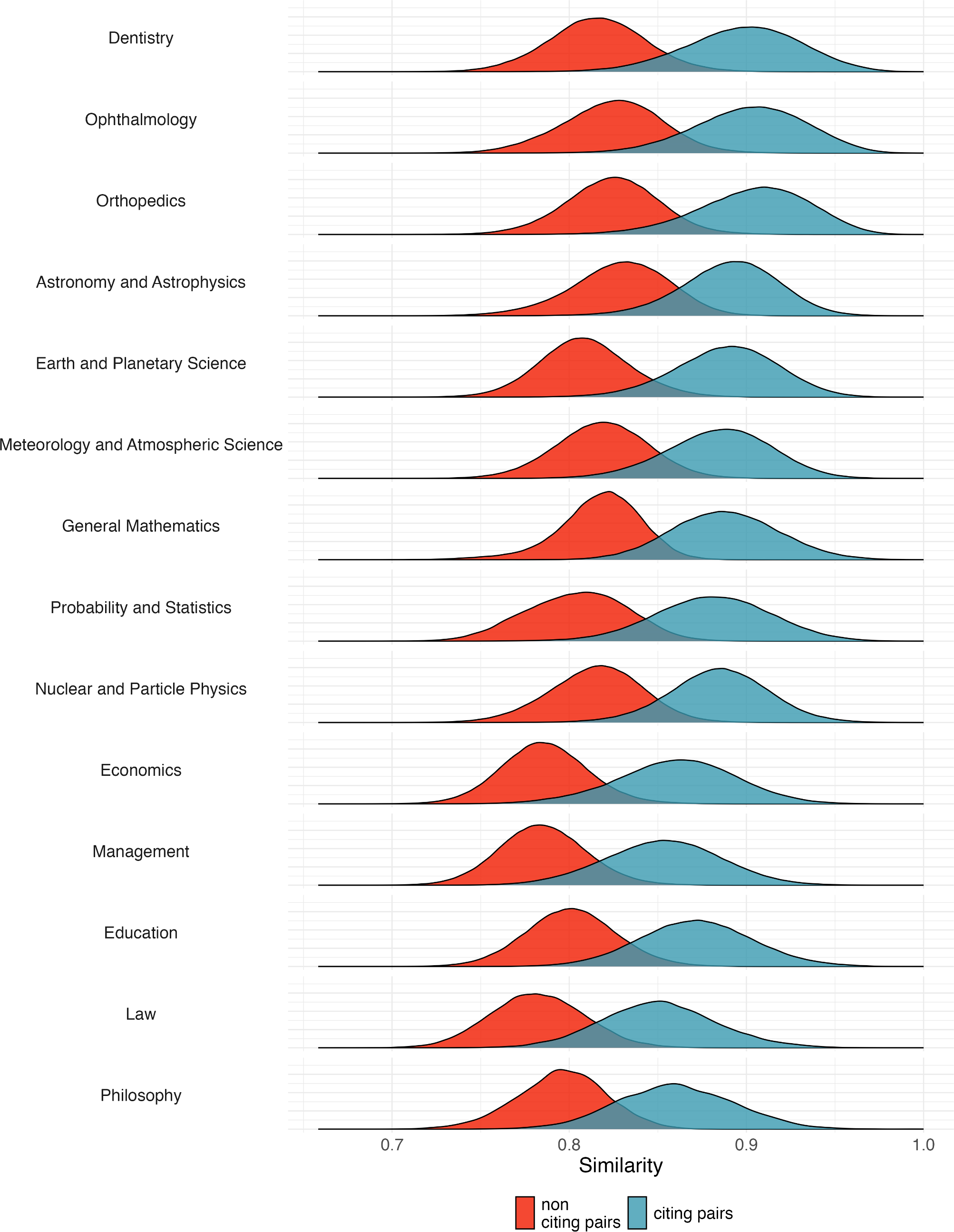

Supplement: S3 Fig — (TIF) [file pone.0335366.s003.tif]

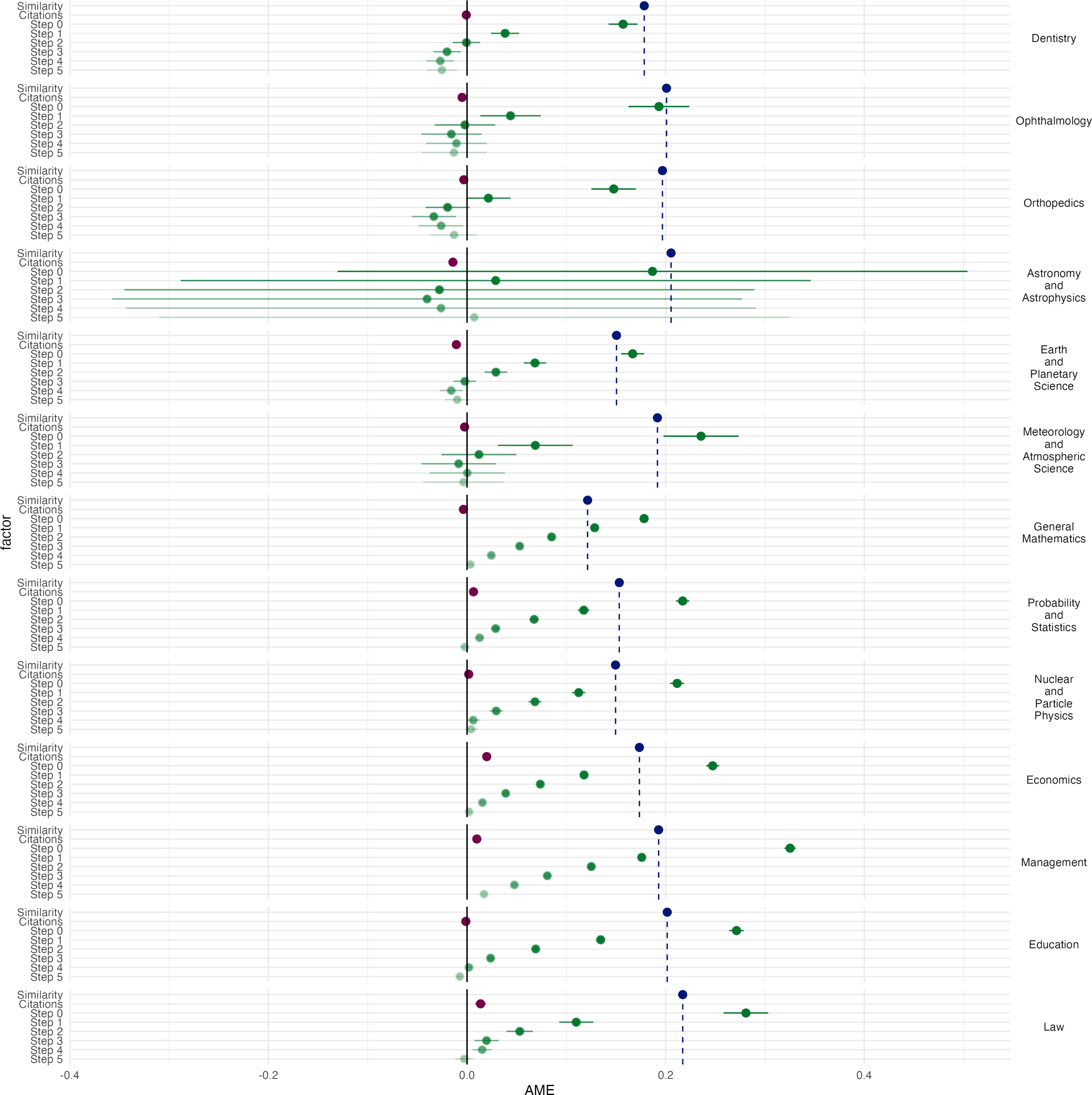

Supplement: S4 Fig — (TIF) [file pone.0335366.s004.tif]

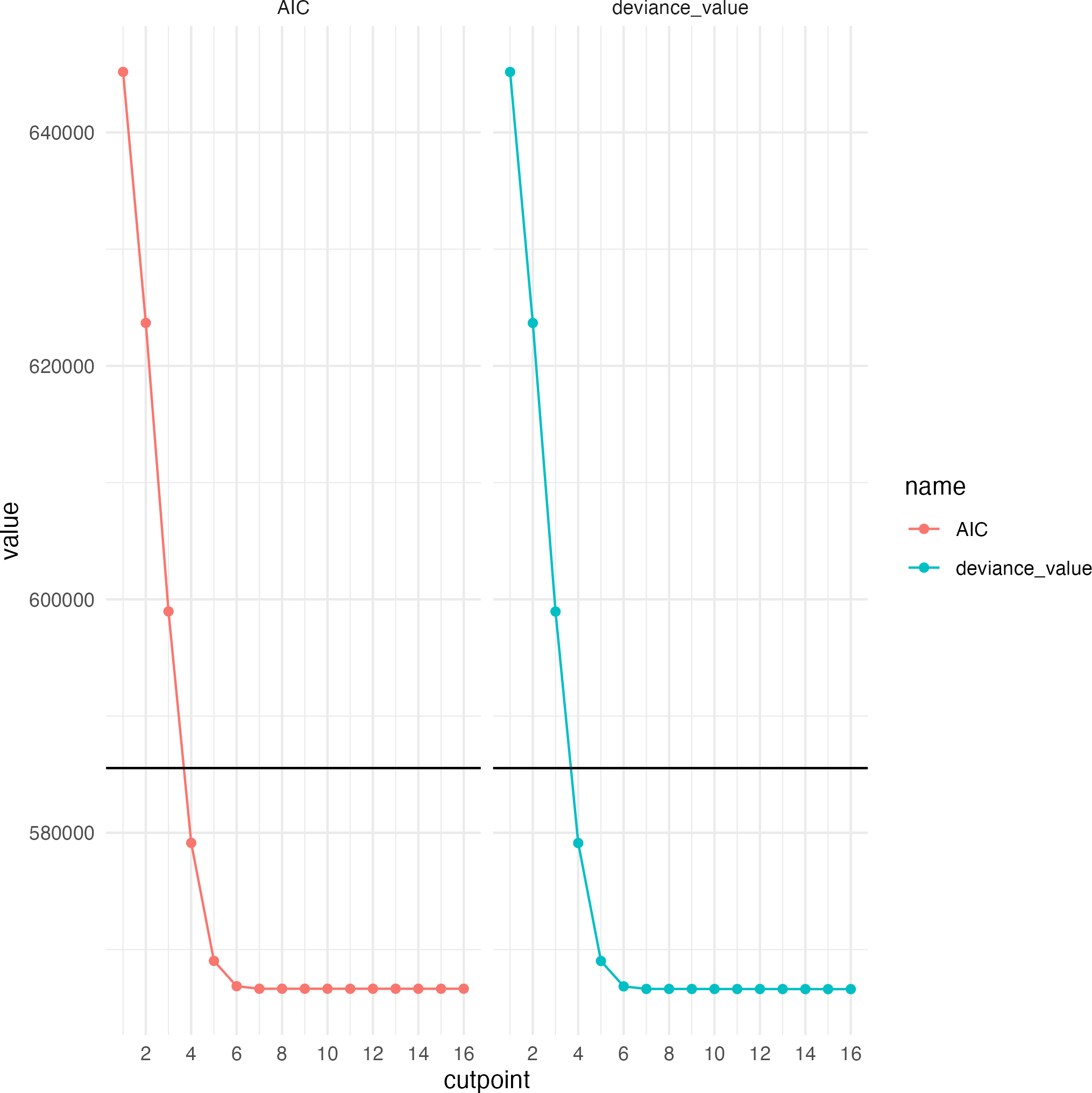

Supplement: S5 Fig — Each cut-point represents a model with that number of dummy variables, where the last category also includes all further distances and is the reference value. The horizontal line represents the model with distance as a continuous variable. Models including only a self-citation flag (cut-point 1), and co-authors flag (cut-point 2) underperform with respect to the continuous model, but models including up to 4–6 degrees of separation show an improvement with respect to the continuous version. After 6, the improvement of the models are marginal. (TIF) [file pone.0335366.s005.tif]

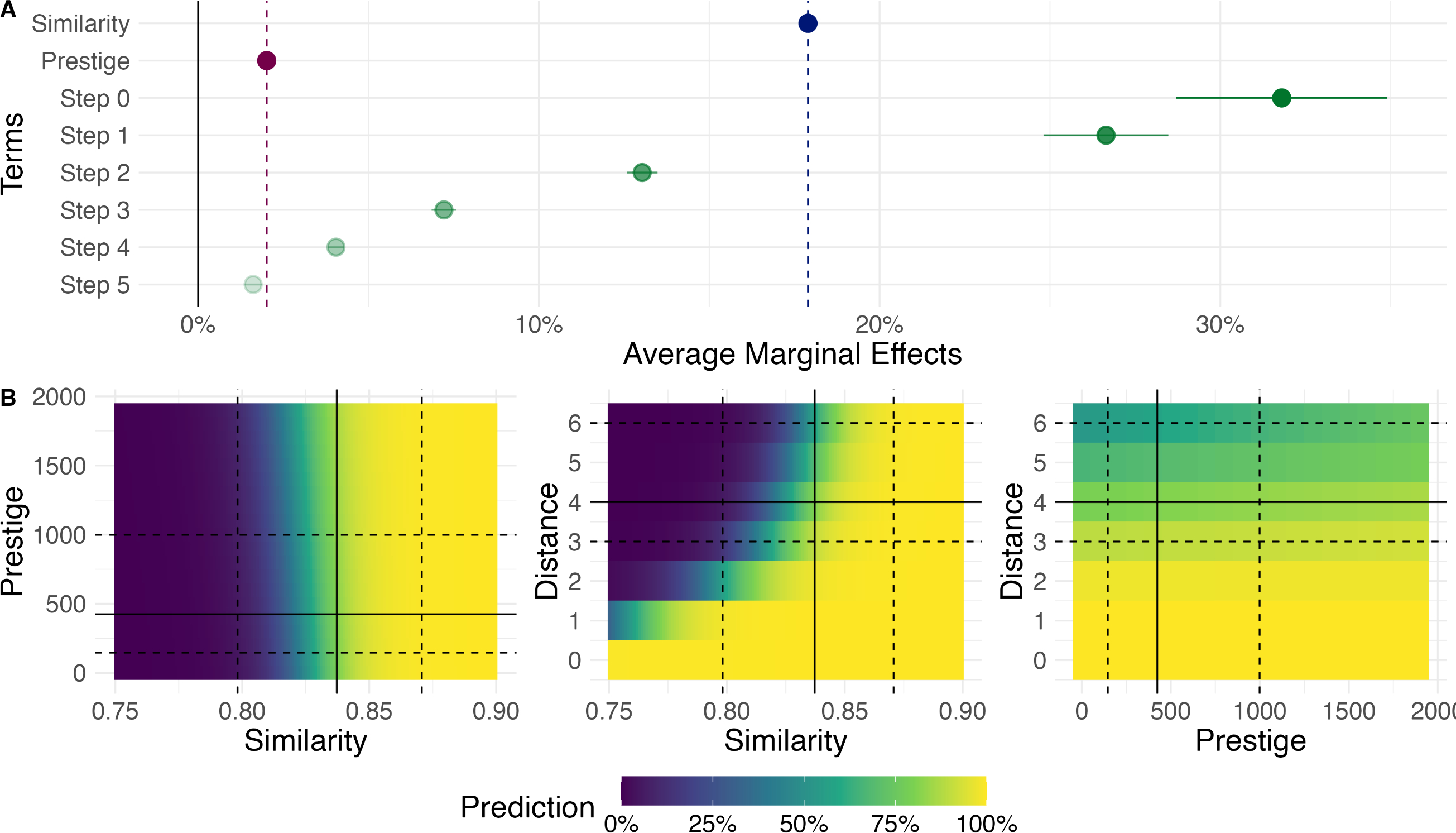

Supplement: S6 Fig — A) Average Marginal Effects of cosine similarity between papers, accumulated citations of cited authors and distances on the collaboration network on the existence of a citation link. B) Predicted probabilities at the interaction of independent variables. Solid lines represent the median of each variable, while dashed lines represent the first and third quartile of their distribution. (TIF) [file pone.0335366.s006.tif]
